# Randomized controlled trial of emotion regulation therapy versus cognitive behavioral therapy to address distress in cancer caregivers

**DOI:** 10.1093/abm/kaag021

**Published:** 2026-05-25

**Authors:** Allison J Applebaum, Jamie M Jacobs, David M Fresco, Michael A Hoyt, Elizabeth Schofield, Morgan J Loschiavo, Mia S O’Toole, Laura E Dunderdale, Douglas S Mennin

**Affiliations:** Brookdale Department of Geriatrics and Palliative Medicine, Icahn School of Medicine at Mount Sinai, New York, NY, 10029, United States; Center for Psychiatric Oncology and Behavioral Sciences, Massachusetts General Brigham Cancer Institute, Boston, MA, 02114, United States; Department of Psychiatry, Harvard Medical School, Boston, MA 02114, United States; Department of Psychiatry & Institute for Social Research, University of Michigan, Ann Arbor, MI, 48109, United States; Department of Population Health and Disease Prevention and the Chao Family Comprehensive Cancer Center, University of California, Irvine, CA, 92697, United States; Department of Psychiatry and Behavioral Sciences, Memorial Sloan Kettering Cancer Center, New York, NY, 10017, United States; Department of Psychiatry and Behavioral Sciences, Memorial Sloan Kettering Cancer Center, New York, NY, 10017, United States; Department of Psychology and Behavioral Sciences, Aarhus University, 8000 Aarhus C, Denmark; Center for Psychiatric Oncology and Behavioral Sciences, Massachusetts General Brigham Cancer Institute, Boston, MA, 02114, United States; Department of Counseling and Clinical Psychology, Teachers College, Columbia University, New York, NY, 10027, United States

**Keywords:** cancer caregivers, caregiver distress, Emotion Regulation Therapy for Cancer Caregivers (ERT-C), Cognitive Behavioral Therapy for Cancer Caregivers (CBT-C), anxiety, depression, quality of life (QOL), caregiver burden

## Abstract

**Background:**

Cognitive Behavioral Therapy (CBT) shows promise for addressing distress in cancer caregivers, though results are mixed. Traditional CBT may not fully address transdiagnostic processes underlying caregiver distress, such as attentional rigidity and perseverative thinking. Emotion Regulation Therapy (ERT), a contemporary CBT targeting these mechanisms, has promise for cancer caregivers (ERT-C). We compared ERT-C to caregiver-adapted CBT (CBT-C) on caregiver- and patient-reported outcomes.

**Methods:**

We conducted a multisite RCT (ERT-C vs. CBT-C) with distressed caregivers of patients with any cancer type or stage. Caregivers completed measures of anxiety, depression, worry, rumination, burden, and quality of life (QOL) at baseline, post-treatment, and 3- and 6-months follow-up. Patients reported outcomes at baseline and 3 months. Linear mixed-effects models with multiple imputation were used to assess group differences.

**Results:**

From March 2021 to April 2024, we randomized 253 caregivers, of whom 244 were analyzed (ERT-C = 124; CBT-C = 120), and enrolled 95 patients (ERT-C = 47; CBT-C = 48), of whom 87 were analyzed. From pre- to post-treatment and through 6 months follow-up, caregivers in ERT-C did not improve significantly more than in CBT-C. Although there were significant between-group differences in patient physical and mental health, these effects did not persist after multiplicity adjustment. However, caregivers overall demonstrated significant improvements over time in anxiety, depression, worry, and QOL, and patients demonstrated significant improvements in perceived stress and emergency room visits.

**Conclusions:**

Although ERT-C was not superior to CBT-C, caregivers showed meaningful psychosocial improvements overall, and patients improved on 2 outcomes. Future work will examine whether the 2 treatments differentially engage putative mechanisms leading to the observed clinical findings.

**Clinical trial registration:**

This trial is registered on ClinicalTrials.gov (identifier: NCT04802720).

## Teaser

For distressed caregivers of patients with cancer, engagement in any evidence-based CBT-informed intervention targeting caregiver-specific distress may be beneficial.

## Introduction

Up to 6 million people in the U.S. serve as family caregivers—partners, parents, siblings, children, extended relatives, and friends—to patients with cancer,^1^ hereafter referred to as “caregivers.” Cancer care increasingly relies on caregivers to shoulder demanding responsibilities^1^^,^^2^ at substantial costs: approximately half report clinically significant depression and/or anxiety,^3–8^ often at higher rates than patients.^3^^,^^4^^,^^9^ Caregivers frequently experience fear of cancer recurrence, a loss of control while witnessing suffering, and difficulties balancing hope with anticipatory grief during cycles of treatment and toxicities.^10–14^ Untreated distress increases over time,^8^^,^^9^ undermining caregivers’ ability to fulfill their essential role on the healthcare team.^15–18^

Cognitive Behavioral Therapy (CBT) has the strongest evidence broadly in patient populations across the lifespan,^19–27^ including dementia caregivers.^19–21^^,^^28–35^ Among cancer caregivers, a growing literature shows some benefits of CBT for anxiety, depression, and insomnia. Our 2017 meta-analysis^36^ of 36 CBT trials showed small effects on psychological, physical, and interpersonal wellbeing (Hedge’s *g*s < 0.20), and a more recent meta-analysis of 17 studies reported moderate effects on depression and anxiety (*g*s = 0.32-.36).^37^ Two trials of CBT integrating acceptance and commitment therapy (ACT) components (eg, acceptance, mindfulness) have shown efficacy among cancer caregivers.^38–41^ However, a recent scoping review of contemporary CBT-based approaches for cancer caregivers reported inconsistent outcomes across studies.^42^ These mixed findings likely reflect heterogeneity in how CBT-based approaches have been defined, adapted, and delivered across caregiver populations.

Emotion Regulation Therapy (ERT)^43^ is a CBT-consistent approach that adds attention-regulation (eg, flexible shifting and sustained attention) and metacognitive regulation (eg, observing thoughts with distance and reinterpreting meaning to shift emotional responses)^44^^,^^45^ together with mindfulness practices and emotional exposure exercises to target internal distress. ERT has established efficacy for chronic anxiety and depression in the general population,^46–48^ and given its targeting of processes that commonly drive caregiver distress,^49–51^ we adapted ERT for cancer caregivers (Emotion-Regulation Therapy for Cancer Caregivers; ERT-C). We subsequently demonstrated ERT-C’s feasibility, acceptability, and promising effects on anxiety, depression, worry, emotion regulation, and perseverative negative thinking in an open trial among caregivers of patients with diverse cancers,^52–54^ and in addition to promising outcomes for caregivers, our initial RCT of ERT-C compared to waitlist control demonstrated improved quality-of-life (QOL) in the patients cared for by enrolled caregivers.^54^ Together, these trials suggested that ERT-C, a contemporary CBT-based approach targeting regulatory mechanisms implicated in caregiver distress, may offer particular benefit for distressed caregivers.

In our overburdened healthcare system and given caregivers’ limited time, efficient, high-yield approaches are needed. No trial has directly compared 2 active, manualized CBT-based psychotherapies for cancer caregivers. ERT-C and CBT-C differ in therapeutic strategies, skill-building targets, and session content,^55^ with ERT-C focused on attentional and metacognitive regulation, and values-guided re-engagement, while CBT is focused on cognitive restructuring, behavioral strategies, psychoeducation, and relaxation. As compared to CBT-C, ERT-C emphasizes homework and between-session skills practice, including the completion of daily monitoring forms. However, the approaches share a common CBT framework and include structured skills training and a focus on caregiver-specific distress. Moreover, while most prior studies of CBT-based approaches for cancer caregivers focused on specific cancers (eg, brain tumors), interventions must be tested across diagnoses to maximize reach and resource efficiency. Additionally, the inconsistent findings to date may also reflect the inclusion of caregivers without baseline distress, whereas robust effects depend on delivering interventions to individuals experiencing caregiving-related distress. To advance the field, we compared the immediate and longer-term efficacy of ERT-C versus traditional CBT for Caregivers (CBT-C) in distressed caregivers across cancer types and stages. We hypothesized that ERT-C would produce greater reductions in anxiety, depression, worry, and rumination (primary outcomes), as well as caregiver quality of life (QOL), burden, and patient-reported quality of life (secondary outcomes).

## Materials and methods

### Design and procedures

This multicenter randomized controlled trial compared the efficacy of ERT-C and CBT-C in improving anxiety, depression, worry, and rumination (primary outcomes), and QOL and caregiver burden (secondary outcomes) among distressed caregivers of patients with varying sites and stages of cancer. A full study design description has been previously published.^55^ Participants completed assessments via REDCap at baseline (T1), within 1 week of the last session (T2), and at 3 (T3) and 6 (T4) months post-treatment. Following consent, caregivers were randomized to ERT-C or CBT-C and met with an interventionist for 8, 1-hour telemedicine sessions, completed within 16 weeks. Caregivers received both printed and digital copies of the intervention manual. Participants also completed a small battery of assessments in advance of each ERT-C/CBT-C session. Patients cared for by participating caregivers were invited to complete measures of QOL, perceived stress, and healthcare utilization at T1 and T3. Caregiver participation was independent of patient participation. Both patients and caregivers received digital gift cards or eChecks as incentives (caregivers received $5 for T1, $10 for each session, $10 for T2, $15 for T3, and $20 for T4; patients received $25 for T3). The study was approved by the IRBs at Memorial Sloan Kettering (IRB #20-407) and Dana-Farber Harvard Cancer Center (IRB #21-074) and registered at ClinicalTrials.gov (NCT04802720).

### Participants and recruitment

Participants were recruited between March 2021 and April 2024 via digital fliers on Memorial Sloan Kettering Cancer Center’s (MSK) and Massachusetts General Hospital’s (MGH) caregiver support pages, clinic schedule screening, and physician referrals.^55^ Eligible caregivers were adults (≥18) of MSK or MGH patients treated within the past year, English-fluent, living in New York, New Jersey, or Massachusetts with any site/stage of cancer who received any type of treatment in the past 12 months whose self-reported Distress Thermometer scores were ≥4, with distress related to caregiving. Exclusion criteria were a history of bipolar disorder, schizophrenia, or schizoaffective disorder, or concurrent psychotherapy that could not be paused. Patients of enrolled caregivers were also invited if aged ≥18, English-fluent, and without severe cognitive impairment; only 1 caregiver per patient was allowed to enroll. All participants provided informed consent.

### Randomization

Participants were randomized 1:1 to ERT-C or CBT-C using the MSK Clinical Research Database’s randomization module, stratified by site (MSK or MGH) with randomly permuted block lengths.

### Interventions

#### Emotion Regulation Therapy for Cancer Caregivers (ERT-C)

ERT-C is a CBT-based psychotherapy targeting early drivers of distress in caregiving—especially threat- and loss-related appraisals and associated unhelpful coping.^56^ It trains caregivers to: (1) detect cues and link them to common response patterns (eg, worry, rumination, self-criticism, reassurance seeking, avoidance/withdrawal, compulsive behaviors); (2) use attentional skills to broaden, shift, and sustain attention under stress; (3) apply reflective thinking to step back from and reframe distressing thoughts; and (4) re-engage in rewarding, valued activities—even amid loss or threat—by practicing these skills in everyday caregiving situations.

#### Cognitive Behavioral Therapy for Cancer Caregivers (CBT-C)

CBT-C is grounded in the cognitive model, which posits that emotional, behavioral, and physiological responses stem from appraisals of situations.^57^ Therapy focuses on modifying cognitions and automatic behaviors to improve emotion regulation and coping. For this study, CBT-C was adapted from prior trials with caregivers to include only traditional CBT components,^38^^,^^39^ with modules on cognitive restructuring, relaxation, and caregiving-specific information.

### Interventionists and adherence to treatment

Interventionists were pre- and post-doctoral clinical psychology externs trained to deliver ERT-C or CBT-C. Training included a half-day workshop and ongoing weekly group supervision to ensure fidelity. Intervention sessions were audio- and video-recorded, and a random subset of 60 cases was independently rated (by MH for CBT-C, MO for ERT-C) for treatment integrity. Raters scored frequency and skillfulness of interventionist actions across all 8 sessions per case. For frequency, ratings ranged from 0 (Interventionist did not address component/engage action) to 2 (Interventionist addressed component/engaged in action in more detail). Skillfulness ratings ranged from 0 (Interventionist was not skillful or performed poorly) to 2 (Interventionist performed action very skillfully).

### Measures

Data were collected between March 2021 and February 2025.

#### Primary outcomes

Anxiety and depression were measured with the Hospital Anxiety and Depression Scale (HADS),^58^ a 14-item questionnaire producing anxiety (HADS-A) and depression (HADS-D) subscales (range: 0-21). The HADS was administered at primary time points and weekly during the course of ERT-C/CBT-C. Worry was measured using the Penn State Worry Questionnaire (PSWQ), a 16-item measure of future oriented trait worry,^59^ and rumination with the Rumination Subscale of the Rumination-Reflection Questionnaire (RRQ), a 12-item measure of perseverative thinking about the past.^60^

#### Secondary outcomes

Caregiver quality of life was measured with the 35-item Caregiver Quality of Life Index-Cancer (CQOL),^61^ and caregiver burden with the 24-item Caregiver Reaction Assessment (CRA), which evaluates self-esteem, family support, finances, and health.^62^

Patients completed the following measures at T1 and T3: the PROMIS Global Health Scale (PROMIS-GH)^63^; EORTC QLQ-C30, a 30-item questionnaire, to assess various domains of quality of life in patients with cancer^64^; the Perceived Stress Scale (PSS), a widely used valid and reliable 9-item measure of the degree to which situations are perceived as stressful^65^; and a 5-item National Survey on Drug Use and Health (NSDUH) module assessing health service utilization over the past 12 months.^66^

### Statistical analysis

The sample was described and dropouts (participants lacking T3 or T4) were compared to full study participants using a series of *t*-tests and Chi-square tests. Immediate effects (T1 to T2) on HADS-A and HADS-D were tested with mixed-effects regression models including time, arm, and their interaction, with random person-level intercepts and adjustment for site. This model incorporated weekly HADS scores during treatment and the parameter of interest was the interaction term. For other immediate outcomes that were not collected weekly during treatment and for maintenance effects (T2 to T4), the change score (T2-T1 for immediate, T4-T2 for maintenance) was regressed on a group indicator with adjustment for site. In mixed-effects models, missing data do not bias results under the missing at random assumption.^67^ However, for modeling of change scores, we employed multiple imputation (MI) via chained equations with a fully conditioned specification model to account for missing data.^68^ We used 20 imputed datasets, with outcomes at all timepoints (T1, T2, T3, T4), clinical site, randomization arm, and any baseline characteristics associated with completion included as auxiliary variables. Fit of the MI was evaluated by convergence and relative efficiency of the variance for the parameter of interest. As an additional exploratory analysis, effect sizes (Cohen’s *d*) with 95% confidence intervals were calculated using Becker’s method and model-adjusted means reported for the overall sample and by arm.^69^ Model-estimated means and effects sizes were extracted directly from the MI results and pooled, using Rubin’s method.^70^ Change scores for patient outcomes were also regressed on arm, with adjustment for site. For one outcome, number of ER visits, a log link for Poisson regression was used due to the measure being a count. While not planned *a priori*, to protect against false discovery due to multiple testing, we adjusted all *P* values for the main parameter across all regression models using the false discovery rate (FDR) method. Treatment completion (all sessions) was compared between arms with Chi-square tests, and reasons for drop-out were summarized. The study was powered with the expectation of enrolling 240 caregivers, 120 patients, and with no greater than 20% attrition. Analyses were conducted in SAS version 9.4.^71^

## Results

### Sample characteristics and attrition

**Figure 1:**
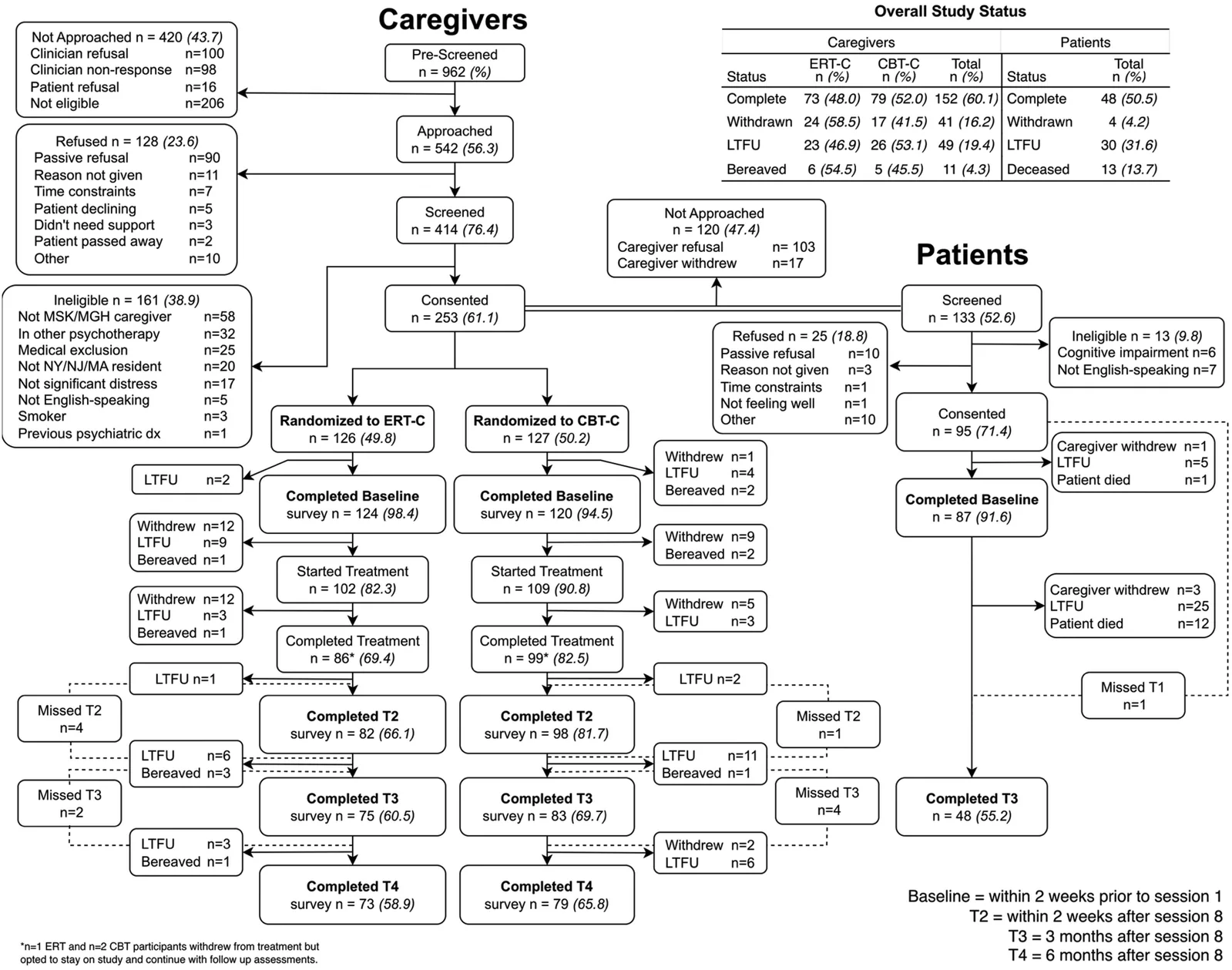
Consort diagram.

Our CONSORT diagram is presented in Figure 1. Of 414 potential participants screened, 253 consented and were randomized, 126 to ERT-C and 127 to CBT-C. Among randomized participants, 244 completed the baseline (T1) assessment, with 124 (51%) assigned to ERT-C. After baseline, 180 caregivers completed the post-treatment assessment (T2), 158 completed the 3-month assessment (T3), and 154 completed the 6-month follow-up assessment (T4). Overall, 80 (32.8%) of the 244 participants with T1 data had no T3 or T4 data, resulting in a 67.2% completion rate. Socioeconomic variables and assignment to condition did not account for differences between completers and non-completers. However, there were differences in psychosocial measures between completers and non-completers. On average, completers as compared to non-completers evidenced a 1.2-point lower HADS anxiety score (*P *= .02), a 10-point lower QOL score (*P* < .001), as well as lower impact of caregiving on their schedule (*P *= .04) and finances (*P *= .003).

Sociodemographic characteristics are presented in Table 1. Participants were, on average, 52.9 years old and predominantly female (72.1%), white (80.7%), partnered (82.4%), and highly educated (52.9% reported completing professional/graduate school). Half of participants (51.2%) worked full time, and the majority (75.8%) reported a high annual income (>$75 000). In terms of caregiving characteristics, on average, caregivers had been in the role for 18.7 months and devoted 7.5 hours/day to caregiving, and the majority (70.5%) were the spouse/partner of the patient. The majority (82.8%) cohabitated with patients. In terms of patient characteristics, the majority had advanced cancer (15.6% Stage III, 42.2% Stage IV), with the most common sites being breast (13.9%) and brain (9.8%) cancer.

**Table 1:** Sample Characteristics of Caregivers (*N* = 244).

Personal characteristics	*n* (%)	Caregiving details	*n* (%)
**Randomization arm: ERT-C**	124 (51%)	Current Caregiver?	
**Gender: female**	176 (72.1%)	No	10 (4.1%)
**Age, M (SD)**	52.9 (13.8)	Yes, constantly	160 (65.6%)
**Race/ethnicity**		Yes, on and off	71 (29.1%)
** African American/Black**	8 (3.3%)	Missing	3 (1.2%)
** Asian/PI**	14 (5.7%)	Time Caregiving (months), M (SD)	18.7 (24.9)
** Caucasian/White**	197 (80.7%)	Hours per day, M (SD)	7.5 (10.3)
** Latino/Hispanic**	10 (4.1%)	Relationship to patient	
** Other/More than 1**	14 (5.7%)	Spouse/partner	172 (70.5%)
** Missing**	1 (0.4%)	Parent	49 (20.1%)
**Religion**		Child	10 (4.1%)
** Catholic**	84 (34.4%)	Sibling	7 (2.9%)
** Protestant**	45 (18.4%)	Other	4 (1.6%)
** Jewish**	39 (16.0%)	Missing	2 (0.8%)
** None**	49 (20.1%)	Live with patient: yes	202 (82.8%)
** Other**	27 (11.1%)	Cancer type	
**Marital status**		Breast	34 (13.9%)
** Married/cohabitating**	201 (82.4%)	Brain	24 (9.8%)
** single**	32 (13.1%)	Colon or rectum	22 (9.0%)
** divorced or separated**	8 (3.3%)	Pancreas	22 (9.0%)
** Widowed**	1 (0.4%)	Multiple myeloma	18 (7.4%)
** Missing**	2 (0.8%)	Lung or bronchus	17 (7.0%)
**Education**		Ovarian	12 (4.9%)
** High school diploma/GED**	6 (2.5%)	Other	95 (38.9%)
** Vocational school or some college**	18 (7.4%)	Cancer stage	
** College degree**	87 (35.7%)	Stage 0 or 1	17 (7.0%)
** Professional or graduate school**	129 (52.9%)	Stage II	21 (8.6%)
** Missing**	4 (1.6%)	Stage III	38 (15.6%)
**HH income**		Stage IV	103 (42.2%)
** Up to $39 999**	9 (3.7%)	Don’t know	65 (26.6%)
** $40 000 to $74 999**	22 (9.0%)	Site	
** $75 000 or more**	185 (75.8%)	MSK	131 (53.7%)
** Missing/prefer not to answer**	28 (11.5%)	MGH	113 (46.3%)
**Employment**		Participant baseline symptoms	
** FT**	125 (51.2%)	Distress (DT), M (SD)	5.5 (2.1)
** PT**	20 (8.2%)	HADS anxiety	
** Self-employed**	19 (7.8%)	M (SD)	11.7 (3.6)
** Retired**	46 (18.9%)	≥8	214 (88.4%)
** Other/not working**	34 (13.9%)	HADS depression	
**Previous medication/therapy**	100 (41.0%)	M (SD)	7.6 (3.5)
**Current medication/therapy**	74 (30.3%)	≥8	119 (49.4%)

### Treatment adherence

In terms of adherence to the treatment manuals, average ratings reflected close interventionist adherence: ERT-C frequency = 1.41, skillfulness = 1.82; CBT-C frequency = 1.71, skillfulness = 1.73. Raters also assessed treatment contamination (ie, elements of ERT-C in CBT-C, and vice versa) and interventionist engagement and rapport skills using a 0-4 scale (0 = Not Present, 4 = Always Present). Average contamination for ERT-C and CBT-C were low at 0.01 and 0.02, respectively. Ratings of interventionist engagement and rapport were high: 4.87 for ERT-C and 4.76 for CBT-C.

### Primary and secondary caregiver outcomes

**Figure 2:**
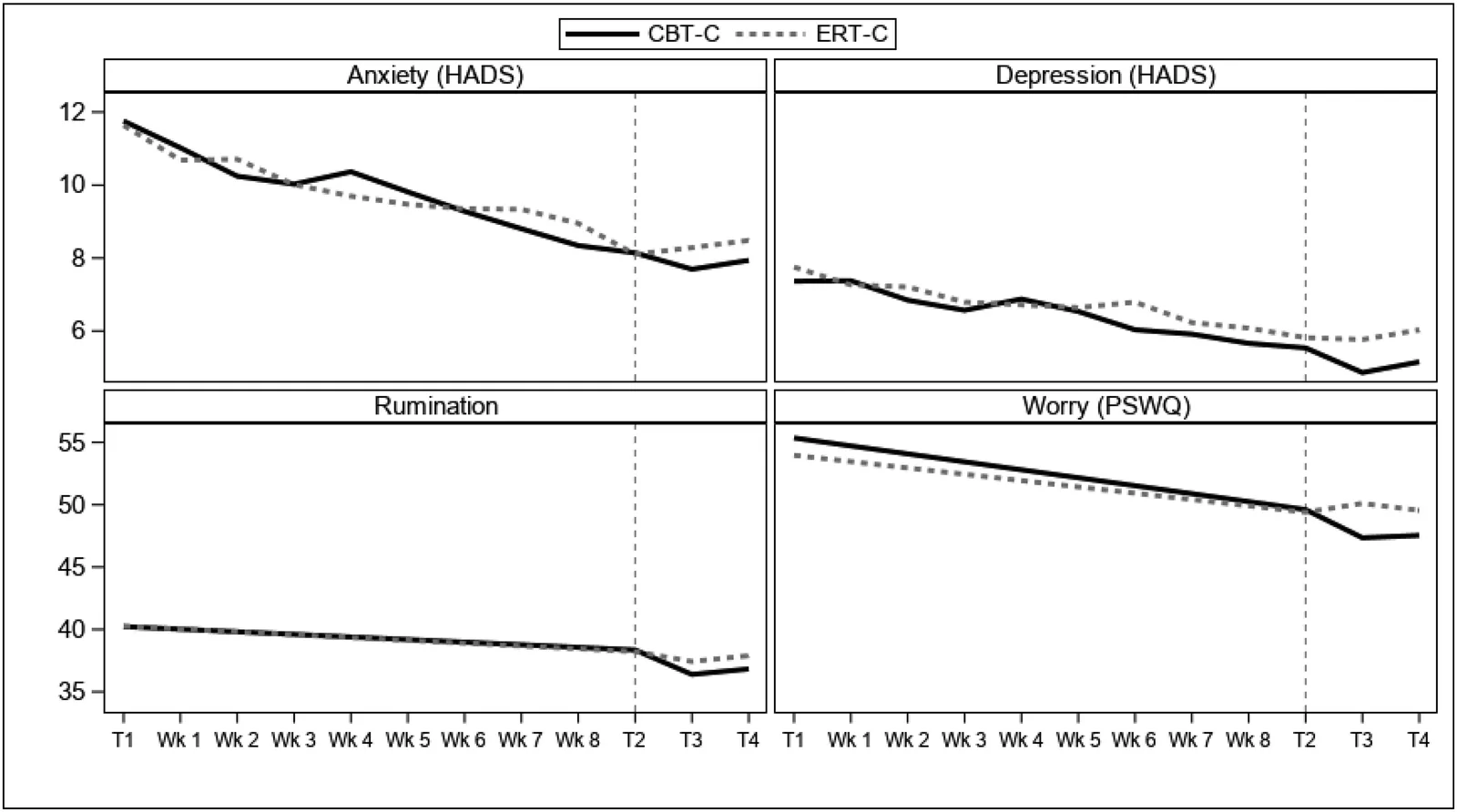
Primary outcomes over time. Immediate effects are assessed between T1 and T2; maintenance effects are assessed between T2 and T4.

As seen in Table 2 and depicted in Figure 2, analyses including weekly data that estimated the parameter of interest, time × group interaction, were not statistically significant (anxiety: *P* = .482; depression: *P* = .330) from baseline through post-treatment (immediate). That is, any improvements in these outcomes did not differ significantly between ERT-C and CBT-C. However, both models did reveal significant main effects of time (anxiety: *b* = −0.30, *P* < .001; depression: *b* = −0.20, *P* < .001).

**Table 2:** Mixed-Effects Modeling Results for HADS Outcomes.

	HADS anxiety		HADS depression	
	*b* (SE)	*P* value	*b* (SE)	*P* value
**Group: ERT-C**	−0.09 (0.43)	.841	0.24 (0.41)	.549
**Time (in weeks)**	−0.32 (0.03)	<.001	−0.20 (0.02)	<.001
**Group × time**	−0.03 (0.04)	.482	0.03 (0.03)	.330
**#Observations**	1869		1868	
**#Participants**	244		244	

HADS anxiety and HADS Depression are regressed in separate mixed-effects models. The outcome is regressed on the group indicator, time (in weeks), and the interaction of group by time. Each model is also adjusted for site and includes a random per-person intercept to account for repeated observations. All *N* = 244 participants with baseline data are included, each with up to 10 assessments between T1 and T2. The tests of the hypotheses are based on the interaction terms for each model.

Models of change scores are summarized in Table 3. Consistent with the primary analytic results, the intercepts in models of the change scores for anxiety and depression were significantly less than zero but neither parameter estimate for the hypothesized randomization arm effect was significant. Two other immediate change scores, worry and caregiver QOL, also had significant intercepts, indicating an overall improvement across arms. However, the main effect of randomization arm was not significant in any of these models. All models across all imputations converged successfully and relative efficiency statistics for the parameter of interest (randomization arm) were all greater than 0.96.

**Table 3:** Regression Modeling Results for Change Scores of Caregiver Outcomes with Multiple Imputation.

	Imputations		Intercept		Arm: ERT-C	
	*n* (%)	Rel. Eff.	*b* (SE)	*P* value	*b* (SE)	*P* value
**Immediate (T1 to T2)**						
** Anxiety (HADS-A)**	65 (27%)	0.98	−3.22 (0.53)	<.001	0.03 (0.64)	.967
** Depression (HADS-D)**	65 (27%)	0.99	−1.39 (0.51)	.006	−0.12 (0.54)	.827
** Worry (PSWQ)**	65 (27%)	0.99	−3.75 (1.35)	.006	0.88 (1.43)	.536
** Rumination (RRQ)**	66 (27%)	0.98	−1.19 (1.08)	.268	−0.21 (1.27)	.867
** Caregiver QOL**	73 (30%)	0.98	−9.80 (2.45)	<.001	1.92 (2.82)	.496
** Caregiver burden**						
** Impact on schedule**	73 (30%)	0.98	0.00 (0.10)	.987	0.01 (0.11)	.897
** Caregiver’s Esteem**	73 (30%)	0.98	0.09 (0.07)	.212	0.07 (0.08)	.396
** Lack family support**	73 (30%)	0.98	−0.14 (0.11)	.223	0.15 (0.12)	.217
** Impact on health**	73 (30%)	0.99	−0.02 (0.08)	.843	−0.06 (0.09)	.545
** Impact on Finances**	74 (30%)	0.98	−0.01 (0.09)	.896	0.11 (0.12)	.345
**Maintenance (T2 to T4)**						
** Anxiety (HADS-A)**	92 (38%)	0.98	−0.16 (0.56)	.775	0.37 (0.67)	.584
** Depression (HADS-D)**	92 (38%)	0.97	−0.25 (0.51)	.627	0.22 (0.67)	.740
** Worry (PSWQ)**	92 (38%)	0.98	−1.06 (1.52)	.489	1.48 (1.86)	.427
** Rumination (RRQ)**	93 (38%)	0.97	−1.14 (1.18)	.336	1.06 (1.73)	.543
** Caregiver QOL**	120 (49%)	0.97	−0.21 (2.75)	.940	−1.01 (3.97)	.801
** Caregiver burden**						
** Impact on schedule**	122 (50%)	0.97	−0.22 (0.17)	.213	0.09 (0.19)	.633
** Caregiver’s esteem**	122 (50%)	0.97	−0.07 (0.09)	.417	0.00 (0.13)	.982
** Lack family support**	122 (50%)	0.98	0.13 (0.11)	.217	−0.13 (0.11)	.245
** Impact on health**	122 (50%)	0.97	−0.09 (0.14)	.524	−0.14 (0.17)	.429
** Impact on finances**	122 (50%)	0.97	0.06 (0.15)	.705	0.16 (0.22)	.454

Each result represents pooled estimates across 20 multiply imputed samples, each with a total of 244 records. Imputation statistics indicate the percentage of observations for which the measure was imputed and relative efficiency (Rel. Eff.) of the multiple imputation on the parameter estimate for randomization arm. All models also include adjustment for site.

The exploratory analysis of effect sizes for change scores includes both overall (aggregating across arms) and by arm. Overall, the greatest improvement immediately was demonstrated in anxiety (M = −3.44, SE = 0.3), which resulted in a large effect size (*d* = −0.86). The other primary outcomes of depression (M = −1.74, SE = 0.3; *d* = −0.47) and worry (M = −4.60, SE = 0.7; *d* = −0.46) demonstrated medium sized effects, and rumination (M = −1.89, SE = 0.6; *d* = −0.24) a small effect. Overall, mean CQOL scores were reduced by over nine points (M = −9.59, SE = 1.4; *d* = −0.55). Of the burden domain scores, the largest effect was seen in Caregiver’s Esteem (M = 0.11, SE < 0.1; *d* = 0.22) which demonstrated a small-medium effect. Standardized effect sizes and model-adjusted means, for both time periods and randomization arms, are given in Table 4.

**Table 4:** Effect Sizes (95% CI) and Model-Adjusted Means (SE) for Change in Outcomes Over Time, by Group.

	Cohens *d* (95% CI)			Adjusted mean (SE)		
	ERT-C	CBT-C	All	ERT-C	CBT-C	All
**Immediate (T1 to T2)**						
** HADS anxiety**	−0.81 (−1.0 to −0.6)	−0.92 (−1.1 to −0.7)	−0.86 (−1.0 to −0.7)	−3.42 (0.4)	−3.45 (0.4)	−3.44 (0.3)
** HADS depression**	−0.47 (−0.7 to −0.3)	−0.46 (−0.6 to −0.3)	−0.47 (−0.6 to −0.3)	−1.80 (0.4)	−1.68 (0.4)	−1.74 (0.3)
** Worry (PSWQ)**	−0.39 (−0.5 to −0.2)	−0.55 (−0.7 to −0.4)	−0.46 (−0.6 to −0.4)	−4.16 (1.1)	−5.05 (1.0)	−4.60 (0.7)
** Rumination (RRQ-R)**	−0.25 (−0.4 to −0.1)	−0.24 (−0.4 to −0.1)	−0.24 (−0.3 to −0.1)	−2.00 (0.9)	−1.79 (0.8)	−1.89 (0.6)
** Caregiver QOL**	−0.45 (−0.6 to −0.3)	−0.66 (−0.8 to −0.5)	−0.55 (−0.7 to −0.4)	−8.63 (2.0)	−10.55 (2.0)	−9.59 (1.4)
**Caregiver burden**						
** Impact on schedule**	−0.05 (−0.2 to 0.1)	−0.06 (−0.2 to 0.1)	−0.05 (−0.2 to 0.1)	−0.03 (0.1)	−0.04 (0.1)	−0.04 (0.1)
** Caregiver’s esteem**	0.27 (0.1 to 0.4)	0.16 (0.0 to 0.3)	0.22 (0.1 to 0.3)	0.14 (0.1)	0.08 (0.1)	0.11 (0.0)
** Lack family support**	0.07 (−0.1 to 0.2)	−0.13 (−0.3 to 0.0)	−0.04 (−0.1 to 0.1)	0.04 (0.1)	−0.11 (0.1)	−0.03 (0.1)
** Impact on health**	−0.15 (−0.3 to −0.0)	−0.07 (−0.2 to 0.1)	−0.12 (−0.2 to −0.0)	−0.10 (0.1)	−0.04 (0.1)	−0.07 (0.1)
** Impact on finances**	0.13 (0.0 to 0.2)	−0.03 (−0.1 to 0.1)	0.06 (−0.0 to 0.1)	0.10 (0.1)	−0.02 (0.1)	0.04 (0.1)
**Maintenance (T2 to T4)**						
** HADS anxiety**	0.08 (−0.1 to 0.2)	−0.01 (−0.2 to 0.1)	0.03 (−0.1 to 0.1)	0.31 (0.5)	−0.06 (0.4)	0.13 (0.3)
** HADS depression**	0.03 (−0.1 to 0.2)	−0.03 (−0.2 to 0.1)	−0.00 (−0.1 to 0.1)	0.10 (0.5)	−0.13 (0.4)	−0.02 (0.3)
** Worry (PSWQ)**	−0.01 (−0.2 to 0.1)	−0.14 (−0.3 to 0.0)	−0.07 (−0.2 to 0.0)	−0.06 (1.4)	−1.55 (1.3)	−0.80 (0.9)
** Rumination (RRQ-R)**	0.01 (−0.1 to 0.2)	−0.10 (−0.3 to 0.0)	−0.04 (−0.1 to 0.1)	0.11 (1.2)	−0.95 (1.1)	−0.42 (0.8)
** Caregiver QOL**	−0.15 (−0.3 to −0.0)	−0.09 (−0.2 to 0.1)	−0.12 (−0.2 to −0.0)	−2.61 (3.0)	−1.60 (2.1)	−2.10 (1.6)
**Caregiver burden**						
** Impact on schedule**	−0.18 (−0.3 to −0.0)	−0.27 (−0.4 to −0.1)	−0.23 (−0.3 to −0.1)	−0.15 (0.1)	−0.24 (0.1)	−0.20 (0.1)
** Caregiver’s esteem**	−0.11 (−0.3 to 0.0)	−0.11 (−0.3 to 0.0)	−0.11 (−0.2 to 0.0)	−0.06 (0.1)	−0.06 (0.1)	−0.06 (0.1)
** Lack family support**	0.01 (−0.1 to 0.1)	0.22 (0.1 to 0.3)	0.11 (0.0 to 0.2)	0.01 (0.1)	0.14 (0.1)	0.07 (0.1)
** Impact on health**	−0.17 (−0.3 to −0.0)	−0.01 (−0.2 to 0.2)	−0.09 (−0.2 to 0.0)	−0.15 (0.1)	−0.01 (0.1)	−0.08 (0.1)
** Impact on finances**	0.09 (−0.1 to 0.2)	−0.08 (−0.2 to 0.1)	0.01 (−0.1 to 0.1)	0.09 (0.1)	−0.07 (0.1)	0.01 (0.1)

Each result represents pooled estimates across 20 multiply imputed samples. Each model is adjusted for site.

**Table 5:** Patient Baseline Descriptives and Regression Modeling Results with Multiple Imputation.

	Baseline		Intercept		Arm: ERT-C	
	Range	M (SD)	*b* (SE)	*P* value	*b* (SE)	*P* value
**PROMIS-GH**						
** Physical (T-score)**	16.2-50.8	34.6 (10.3)	−0.50 (1.9)	0.795	6.12 (2.4)	0.013^a^
** Mental (T-score)**	21.2-53.3	38.4 (9.6)	−0.52 (1.7)	0.767	5.70 (2.2)	0.011^b^
**EORTC-QLQ30**						
** Global health**	0-100	46.8 (17.7)	−2.70 (3.9)	0.492	−6.61 (6.0)	0.275
**Perceived stress (PSS)**	3-40	17.7 (7.6)	−2.69 (1.2)	0.031	−0.26 (1.7)	0.878
**Healthcare utilization (past 12 m)**						
** ER visits^b^**	0-6	1.2 (1.4)	−1.32 (0.5)	0.006	−0.11 (0.7)	0.865

Each patient outcome is modeled separately, with the T3 change score regressed on randomization arm and adjusted for site. Table represents pooled results over 20 multiply imputed datasets.

a The finding did not remain significant after FDR adjustment.

b The model for number of ER visits is based on a Poisson regression with log transform of the change score.

### Patient outcomes

Among the 244 eligible patients of enrolled caregivers, 87 (36%) enrolled and completed T1. At baseline, patients exhibited scores near the midpoint for physical (M = 34.6, SD = 10.3), mental (M = 38.4, SD = 9.6), and global health scores (M = 46.8, SD = 17.7) as well as for perceived stress (M = 17.7, SD = 7.6; see Table 5). On average, patients had around one ER visit in the previous year (M = 1.2, SD = 1.4). About half (*n* = 47, 54%) of enrolled patients completed the T3 assessment. Follow-up completion was not statistically associated with baseline patient demographics or study measures, but several baseline measures (ie, physical health *P* = .050, mental health *P* = .053, and global health *P* = .061) trended toward significance in the direction of completers having better baseline values. Overall, the sample significantly improved in perceived stress and number of ER visits, as seen via the model intercepts. As with caregiver measures, a complete case analysis found that none of the patient change scores differed significantly by randomization arm, thus providing no support for the hypotheses. When MI was used to handle missing data, changes in both physical (*b* = 6.12, *P* = .013) and mental health (*b* = 5.70, *P* = .011) were significantly higher in the ERT-C arm. These findings of a group effect did not, however, hold up after FDR adjustment for multiplicity (adjusted *P* = .163 for both).

### Treatment completion

A significantly greater number of caregivers randomized to CBT-C versus ERT-C completed treatment (99 versus 86, *P* < .02). However, when excluding individuals who never initiated treatment (ie, attended no sessions), the difference in completion rates between arms was not statistically significant. Among caregivers who attended at least one session of ERT-C or CBT-C, retention rates were comparable across arms. Commonly reported reasons for withdrawal or non-initiation included time constraints (*N* = 12 assigned to ERT-C, *N* = 8 assigned to CBT-C) and the therapy not being a good fit (*N* = 6 assigned to ERT-C, *N* = 2 assigned to CBT-C).

## Discussion

This multicenter RCT is among the largest trials comparing 2 active, manualized psychotherapies for distressed cancer caregivers. Both ERT-C and CBT-C produced clinically meaningful reductions in anxiety (Cohen’s *d* = 0.86), depression (*d* = 0.47), and worry (*d* = 0.46), along with improvements in caregiver quality of life from pre- to post-treatment. However, our *a priori* hypothesis that ERT-C would outperform CBT-C was not supported; the two conditions did not differ significantly on any primary or secondary outcome at any time point. The effect sizes reported here correspond favorably to findings from prior meta-analyses.^36^^,^^37^ Although we anticipated that ERT-C’s attention and metacognitive skills training would yield greater improvement than a traditional CBT package, our results highlight a critical lesson: for distressed cancer caregivers, engagement in any evidence-based CBT-informed intervention targeting caregiver-specific distress may be beneficial.

While the predicted group differences were not detected, two patient outcomes improved across the full sample: perceived stress and ER utilization. This pattern aligns with a prior RCT comparing ERT-C to a waitlist control, in which patients of caregivers randomized to ERT-C reported gains in QOL despite no significant change in their psychological distress.^54^ These findings reinforce the buffering effect of caregiver mental health on patient overall health and emotional functioning, and highlight the downstream effects of caregiver engagement in empirically supported approaches on patients and the healthcare system.

In terms of attrition, non-completers (32.8% of individuals providing baseline data) reported higher baseline anxiety, worse quality of life, and greater financial burden than completers, suggesting that the most distressed caregivers faced significant challenges to engagement. However, the between-arm difference in treatment completion was driven by caregivers who never initiated treatment; completion rates did not differ by condition among individuals who attended at least one session. While multiple imputation addressed missing data under MAR assumptions, the clinical profile of non-completers warrants caution about the completeness of that assumption. Importantly, the willingness of all eligible caregivers screened to enroll during COVID-19 lockdown, and the successful recruitment of more caregivers (*n *= 253) than planned (*n *= 200), underscores the urgency of delivering psychosocial care and the feasibility of targeted, time-limited telehealth support.

Despite unsupported hypotheses, the trial demonstrates the benefit of empirically supported therapeutic components embedded in these CBT-based packages to address distress across diagnoses and the caregiving trajectory. Indeed, the overall effects may have been driven, in part, by common factors, including therapeutic alliance, expectancy for improvement, manualization, structured skill training, the caregiver-specific focus, and weekly contact.^72^ Convergence at the symptom level does not, however, preclude the possibility that the two treatments engage distinct change processes, and planned secondary analyses will examine differential mechanisms of action in treatment-engaged participants.

Although we found significant improvements overall, the absence of a no-treatment or waitlist control arm means that pre-post changes may partially reflect a natural recovery trajectory, and we cannot isolate the contribution of the interventions from passage of time or regression to the mean. Another limitation is the restricted sociodemographic profile of participants. Future trials should include a non-active or non-CBT comparator, recruit more ethno-racially and geographically diverse caregivers, and evaluate delivery models that address time constraints and fit of treatment. Studies are also needed to address scalability beyond NCI-designated comprehensive cancer centers and delivery by a wider group of healthcare professionals. Finally, future work should examine cultural and linguistic adaptations (eg, delivery in Spanish), and the use of asynchronous or digital therapeutic platforms. Overall, our findings underscore the benefits of caregiver engagement in CBT-based approaches to care for caregivers *and* patients and highlight the promise of both ERT-C and CBT-C to meet the psychosocial needs of distressed cancer caregivers.

## Data Availability

The data that support the findings of this study are available from the corresponding author upon reasonable request.
